# Improved Prediction of Endoxifen Metabolism by CYP2D6 Genotype in Breast Cancer Patients Treated with Tamoxifen

**DOI:** 10.3389/fphar.2017.00582

**Published:** 2017-08-24

**Authors:** Werner Schroth, Stefan Winter, Thomas Mürdter, Elke Schaeffeler, Diana Eccles, Bryony Eccles, Balram Chowbay, Chiea C. Khor, Arafat Tfayli, Nathalie K. Zgheib, Michel Eichelbaum, Matthias Schwab, Hiltrud Brauch

**Affiliations:** ^1^Dr. Margarete Fischer-Bosch-Institute of Clinical Pharmacology Stuttgart, Germany; ^2^Department of Clinical Pharmacology, University of Tübingen Tübingen, Germany; ^3^Cancer Sciences Academic Unit and University of Southampton Clinical Trials Unit, Faculty of Medicine, University of Southampton Southampton, United Kingdom; ^4^Dorset Cancer Centre Poole, United Kingdom; ^5^Laboratory of Clinical Pharmacology, Division of Medical Sciences, National Cancer Centre Singapore, Singapore; ^6^Clinical Pharmacology, SingHealth Singapore, Singapore; ^7^Office of Clinical Sciences, Duke-NUS Medical School Singapore, Singapore; ^8^Division of Human Genetics, Genome Institute of Singapore Singapore, Singapore; ^9^Singapore Eye Research Institute Singapore, Singapore; ^10^Department of Ophthalmology, Yong Loo Lin School of Medicine, National University of Singapore Singapore, Singapore; ^11^Hematology-Oncology Division, Department of Internal Medicine, Faculty of Medicine, American University of Beirut Beirut, Lebanon; ^12^Department of Pharmacology and Toxicology, Faculty of Medicine, American University of Beirut Beirut, Lebanon; ^13^Discipline of Pharmacology, Adelaide Medical School, Faculty of Health and Medical Sciences, The University of Adelaide, Adelaide SA, Australia; ^14^Department of Clinical Pharmacology, Institute of Experimental and Clinical Pharmacology and Toxicology, University Hospital Tübingen Tübingen, Germany; ^15^Department of Pharmacy and Biochemistry, University of Tübingen Tübingen, Germany; ^16^German Cancer Consortium of German Cancer Research Center Heidelberg, Germany

**Keywords:** endoxifen, CYP2D6 polymorphism, metabolizer phenotype, tamoxifen, breast cancer

## Abstract

**Purpose:** Prediction of impaired tamoxifen (TAM) to endoxifen metabolism may be relevant to improve breast cancer treatment, e.g., via TAM dose increase. The polymorphic cytochrome P450 2D6 (CYP2D6) strongly determines an individual’s capacity for endoxifen formation, however, CYP2D6 phenotype assignments inferred from genotype widely differ between studies. Thus, we modeled plasma endoxifen predictability depending on variable CYP2D6 genotype groupings.

**Methods:** CYP2D6 diplotype and metabolite plasma concentrations were assessed in 908 pre- and post-menopausal estrogen receptor (ER)-positive, TAM treated early breast cancer patients of Caucasian (*N* = 678), Middle-Eastern Arab (*N* = 77), and Asian (*N* = 153) origin. Robust coefficients of determination (*R*^2^) were estimated for endoxifen (E) or metabolic ratio endoxifen/desmethyl-TAM (E/DMT) as dependent and different CYP2D6 phenotype assignments as independent variables. Allele activity scores (ASs) were modified with respect to a reduced ^∗^10 allele activity. Predictability of endoxifen plasma concentrations above the clinical threshold of 5.9 ng/mL was investigated by receiver operating characteristic (ROC) analysis.

**Results:** CYP2D6 diplotypes (*N* = 898) were strongly associated with E and E/DMT independent of age (*P* < 10^-15^). Across all ethnicities, 68–82% inter-patient variability of E/DMT was explained by CYP2D6 diplotype, while plasma endoxifen was predictable by 39–58%. The previously used codeine specific phenotype classification showed worse prediction for both endpoints particularly in Asians (median *R*^2^< 20%; *P* < 10^-9^). Downgrading of ^∗^10 activity slightly improved the explanatory value of metabolizer phenotype (*P* < 0.002). Endoxifen plasma concentrations above the clinical threshold of 5.9 ng/mL were achieved in 82.3% of patients and were predictable (96% sensitivity, 57% specificity) by CYP2D6 diplotypes with AS > 0.5, i.e., omitting PM/PM and PM/IM patients.

**Conclusion:** The CYP2D6 explanatory power for active drug level assessment is maximized by TAM-specific phenotype assignments while a genotype cutoff that separates PM/PM and PM/IM from the remaining patients may improve clinical benefit via increased endoxifen concentrations.

## Introduction

Tamoxifen (TAM) is a widely prescribed antiestrogen for the control of estrogen receptor (ER)-positive breast cancer, yet its efficacy is reduced due to the development of endocrine resistance and intrinsic patient characteristics that prevent drug response. The latter has been partially attributed to a lack of TAM bioactivation toward its active metabolite, endoxifen. Pharmacological and pharmacogenetic evidence strongly support that *in vivo* endoxifen formation is mainly mediated from the primary metabolite *N*-desmethyl-TAM by the cytochrome P450 2D6 (CYP2D6) enzyme (Stearns et al., 2003; Desta et al., 2004). As distinct genetically determined functional variants are present in the general population, inter-patient variability of plasma endoxifen is expected to be predictable, at least in part, by *CYP2D6* (de Vries Schultink et al., 2015).

The *CYP2D6* polymorphism with more than 100 known alleles contributes to inter-individual differences in enzyme activities and plasma exposure of metabolized drugs and are commonly grouped into four CYP2D6 metabolizer phenotypes: ultra-rapid (UM), extensive (EM), intermediate (IM), and poor (PM) metabolizers. Traditionally, these have been defined using probe substrates, however, due to probe drug differences to derive phenotypes, CYP2D6 genotyping has emerged as the method of choice to predict enzyme activity (Hicks et al., 2014). Activity scores (ASs) of 0, 0.5, and 1 for null (PM), reduced-function (IM), and fully-functional (EM) alleles, respectively, have been used to infer metabolizer phenotypes from diplotypes (Gaedigk et al., 2008). Of note, there is no universally accepted method to assign allele ASs that reflect metabolic activity across all enzyme substrates. In particular, this refers to the activities of reduced-function alleles ^∗^9, ^∗^10, ^∗^17, ^∗^29, and ^∗^41, of which ^∗^10 has been suggested to have a more deleterious effect on enzyme function compared to the remaining IM alleles (Steimer et al., 2004; Shen et al., 2007). A recent literature review across various CYP2D6 substrates suggested that a ^∗^10 AS downgrade from 0.5 to 0.25 may better reflect the functional impairment in ^∗^10 defined IM individuals (Hicks et al., 2014).

Prediction of an impaired TAM metabolizer phenotype (IM, PM) with low endoxifen formation capacity is potentially important for personalized treatment decisions in breast cancer such as increasing the therapeutic TAM dose or replacing TAM with an aromatase inhibitor (AI). Although prospective data that demonstrate a clinical benefit of dose adjustment are lacking, a retrospectively defined clinical threshold of 5.9 ng/mL (15.8 nM) plasma endoxifen separated patients below this cutoff into those with reduced clinical benefit from the remainders (Madlensky et al., 2011; Saladores et al., 2015), suggesting a clinical relevance of predicting endoxifen formation capacity. Since routine therapeutic endoxifen monitoring is not standard clinical practice, genotyping has been put forward and tested in several studies as a prospective tool to select patients for TAM dose escalation or to establish its predictive value (Irvin et al., 2011; Kiyotani et al., 2012; Dezentjé et al., 2015; Hertz et al., 2015; Fox et al., 2016). In the absence of standardized guidelines (Hicks et al., 2014), studies used different phenotype assignments including that based on CYP2D6-dependent codeine metabolism (Crews et al., 2014). As a consequence, low endoxifen predictability from codeine-specific CYP2D6 phenotype assessment in a recent study (Fox et al., 2016) led to recommendations against the use of CYP2D6 genotype to guide clinical decisions (Hertz and Rae, 2016a).

While the combination of multi-locus genotypes into diplotypes based on the AS system (Gaedigk et al., 2008) appears straightforward, their attribution to a specific metabolizer phenotype has been inconsistent preventing meaningful clinical conclusions. Here, we used different metabolizer phenotype definitions to test the power of CYP2D6 diplotype and phenotype-based prediction of impaired endoxifen metabolism with the goal to provide a robust algorithm toward the standardization of CYP2D6 in personalized endocrine treatment.

## Materials and Methods

### Patients

The genotype data and available TAM and TAM metabolite concentrations of 908 prospectively recruited ER-positive breast cancer patients that had received adjuvant TAM treatment (20 mg/d) for at least 6 months and who had TAM plasma concentrations above 150 nM as a threshold for compliance (Saladores et al., 2015) were included in this study. Patients include 367 post-menopausal Caucasian women derived from a German observational trial of outcome predictors in adjuvant endocrine treatment (DRKS 00000605) that were extended from Mürdter et al. (2011), and three ethnic groups of premenopausal Caucasian, Asian, and Middle-Eastern Arab women (*N* = 541) as previously described (Saladores et al., 2015). The rate of patients taking strong CYP2D6 inhibitors was <1% in post-menopausal Caucasians, absent (Asians, Middle-Eastern Arab), or unknown (premenopausal Caucasians). This study was carried out as previously described in accordance with the recommendations of the Ethics Review Committee University of Tübingen, National Cancer Centre Ethics Review Committee (Singapore), American University of Beirut Institutional Review Board (Lebanon) and South and West MultiCentre Research Ethics Committee (MREC 00/6/69; POSH) with written informed consent from all subjects. All subjects gave written informed consent in accordance with the Declaration of Helsinki.

### Genotyping, Phenotype Definition, and Plasma Metabolite Measurement

CYP2D6 diplotypes were assessed in 898 patients by alleles predictive of metabolizer status PM (^∗^3, ^∗^4, ^∗^5, ^∗^6, ^∗^7), IM (^∗^9, ^∗^10, ^∗^41), EM (absence of variant alleles, or ^∗^1, ^∗^2, ^∗^35) and ultra-rapid, UM (duplicated EM allele) with ASs 0, 0.5, 1, and 2, respectively per allele (Gaedigk et al., 2008). Genotyping of variant alleles was done from blood-derived germline DNA based on certified and validated platforms: INFINITI ^TM^ (Autogenomics) was used for the Asian cohort (Lim et al., 2011) and matrix-assisted, laser desorption/ionization, mass spectrometry and TaqMan allelic discrimination assays (Applied Biosystems, Foster City, CA, United States) that infer EM status by the absence of variant alleles were used for the remaining patients (Schroth et al., 2010; Mürdter et al., 2011; Saladores et al., 2015). CYP2D6 gene deletion (^∗^5) and duplications were determined via TaqMan Copy Number Assay (Applied Biosystems) and patients with gene duplications and the absence of variant alleles were assigned UM. For quality assurance, a total of 39 genotypes (4.3%) with an ambiguous duplication status or genotype was verified by AmpliChip P450 assay (Roche Molecular Diagnostics, Mannheim, Germany) thereby discriminating patients with duplication of functional alleles (UM) from duplications in the presence of variant alleles (non-UM).

As a reflection of the heterogeneity of previous TAM dose escalation studies in regard to binning CYP2D6 diplotypes into phenotypes, and to test new hypotheses, CYP2D6 phenotypes were defined as follows (**Table 1**): “Codeine” – the codeine metabolism-based grouping defined by the Clinical Pharmacogenetics Implementation Consortium (Crews et al., 2014) is one of the most frequently used CYP2D6 classification and have been used in a recent TAM dose escalation trial (Fox et al., 2016); “TAM1” – TAM specific phenotype assignment used in TAM intervention trials (Irvin et al., 2011; Hertz et al., 2015); “TAM2” – newly proposed TAM specific phenotype assignment which, based on the distribution of plasma endoxifen concentrations in this study, suggests the binning of EM/IM into EM and of IM/PM into PM; “TAM3” – newly proposed TAM specific phenotype assignment which applies a downgrade of ^∗^10 activity (Hicks et al., 2014) by separating ^∗^10 (AS 0.25) from other IM alleles (AS 0.5); “TAM4” – newly proposed TAM specific phenotype assignment that extends the ^∗^10 allele downgrade as in TAM3 by further downgrading ^∗^10 containing IM genotypes into a new *slow metabolizer* (SM) group.

**Table 1:** CYP2D6 diplotypes with activity scores (ASs) and observed frequencies, and five evaluated phenotypic groupings for the prediction of plasma endoxifen metabolizer status.

Diplotype	AS^a^	N (898)	%	Codeine	TAM1	TAM2	TAM3^c^	TAM4^d^
EM/UM	3	18	2.0	UM	UM	UM	UM	UM
EM/EM	2	300	33.4	EM	EM	EM	EM	EM
EM/IM	1.5	168	18.7	EM	IM	EM	EM	EM
*EM/^∗^10^b^*	*1.25*	*60*	*6.7*	–	–	–	IM	EM
EM/PM	1	221	24.6	EM	IM	IM	IM	IM
IM/IM	1	68	7.6	EM	IM	IM	IM	IM
*^∗^10/^∗^10^b^*	*0.5*	*45*	*5.0*	–	–	–	IM	SM
IM/PM	0.5	73	8.1	IM	IM	PM	IM	SM
*PM/^∗^10^b^*	*0.25*	*19*	*2.1*				PM	SM
PM/PM	0	50	5.6	PM	PM	PM	PM	PM

*^a^Calculated as sum of allele activities for PM (0), IM (0.5), EM (1), and UM (2) as described in Gaedigk et al. (2008); note, in ROC analyses EM/PM and IM/IM were distinguished assuming activities of 1 and 0.75, respectively. ^b^For adjusted IM phenotype definitions ^∗^10 AS was reduced from 0.5 to 0.25 in TAM3 and TAM4. ^c^Reduced ^∗^10 activity with diplotype AS of 1.5–2 (EM), 0.5–1.25 (IM), and 0–0.25 (PM). ^d^Reduced ^∗^10 activity and definition of a slow metabolizer (SM) group with diplotype AS of 1.25–2 (EM), 1 (IM), 0.25–0.5 (SM), and 0 (PM). Diplotype categories: UM, ultra-rapid-; EM, extensive-; IM, intermediate-; SM, slow-; PM, poor- metabolizer; AS, activity score.*

Data of TAM and its metabolites *N*-desmethyl-TAM (DMT) and (Z)-endoxifen were taken (Mürdter et al., 2011; Saladores et al., 2015) with extended numbers of post-menopausal patients plasma that were measured by liquid chromatography tandem mass spectrometry as described (Mürdter et al., 2011). To account for alternative and upstream pathways of endoxifen formation from (Z)-4-hydroxy-TAM and *N-*desmethyl-TAM, CYP2C9^∗^2 and ^∗^3 alleles exerting decreased enzyme function, as well as CYP3A5^∗^3 encoding a non-functional protein were genotyped as previously described (Mürdter et al., 2011; Saladores et al., 2015).

### Statistical Analysis

Endoxifen concentration (E) or metabolic ratio E/DMT were transformed as previously described (Saladores et al., 2015). The effect of CYP2D6 diplotypes and phenotype classifications on E and E/DMT was assessed by linear modeling in 879 patients with available CYP2C9^∗^2, ^∗^3 and CYP3A5^∗^3 genotypes as covariates. Robust adjusted coefficients of determination (*R*^2^) and 95% confidence intervals (CI) were estimated based on 10.000 bootstrap replicates using R-3.3.2^1^ package robustbase_0.92-7 (Rousseeuw et al., 2016). Analysis of deviance was applied to test between linear models including one and two CYP2D6 phenotype assignments as independent variables, respectively. The specificity and sensitivity of CYP2D6 diplotypes in predicting an endoxifen plasma concentration above the clinical threshold of 5.9 ng/mL was investigated by receiver operating characteristic (ROC) curves, using R-package pROC_1.9.1 (Robin et al., 2011). In this analysis, diplotype specific CYP2D6 activities were ordered as follows: EM/UM > EM/EM > EM/IM > EM/PM > IM/IM > PM/IM > PM/PM (this order is identical to the AS, except that EM/PM and IM/IM are distinguished assuming a lower activity of the latter). Estimates of 95% confidence intervals for specificity, sensitivity, and false discovery rate (FDR) were based on 10,000 bootstrap replicates, and the reported measures were selected based on the maximization of Youden’s index.

## Results

### CYP2D6 Activity and Effect of Covariates

**FIGURE 1:**
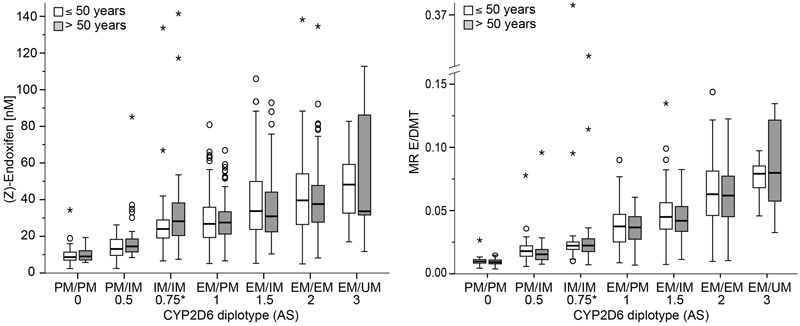
Patient plasma concentrations of (Z)-endoxifen **(left)** and metabolic ratio (Z)-endoxifen/desmethyl-TAM (E/DMT, **right**) depending on CYP2D6 diplotype and age in 897 patients. Concentrations are presented as Tukey boxplots with mild (circle) and extreme (asterisk) outliers. Numbers below diplotypes refer to their respective activity score (AS). ^∗^Note, EM/PM and IM/IM which both sum to AS = 1 according to the Gaedigk et al. (2008) system were distinguished in the current study by assuming a value between 0.5 and 1 for IM/IM.

There was a strong association between CYP2D6 diplotype/AS and endoxifen concentrations or metabolic ratio E/DMT across all patients (**Figure 1**; *P* < 10^-15^). The distribution of E and E/DMT depending on diplotype did not differ between subgroups of women younger or older than 50 years, indicating an identical TAM metabolism irrespective of age or menopausal status. While the median CYP2D6 activity (based on E and E/DMT ratio) increased monotonically with increasing AS, the range of phenotypic activity was smaller in patients with severely impaired activity (AS ≤ 0.5). Specifically, 45 out of 50 PM/PM patients (90%) had low endoxifen based on a proposed threshold of 5.9 ng/mL (Madlensky et al., 2011). Conversely, there was a greater variability in patients with AS ≥ 1 (E/DMT) with several outliers that strongly differed to expected CYP2D6 function.

Wilcoxon-Mann-Whitney tests revealed a significant median reduction of 12% for DMT/TAM or a 26% reduction for 4-OH-TAM/TAM metabolic ratios when comparing CYP3A5^∗^3 or CYP2C9^∗^2/^∗^3 homozygotes to their respective functional ^∗^1/^∗^1 genotype. Thus, both pharmacogenes were included as covariates in the linear modeling.

### CYP2D6 Phenotype Modeling

**FIGURE 2:**
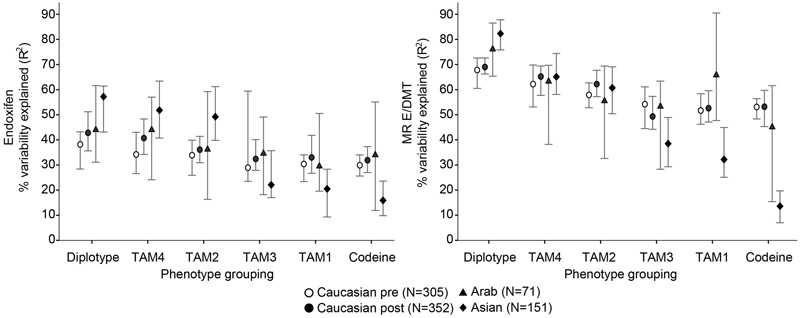
Explained variability of (Z)-endoxifen **(left)** and metabolic ratio (Z)-endoxifen/desmethyl-TAM (E/DMT, **right**) according to different CYP2D6 phenotype classifications inferred by diplotype in 879 patients of three different ethnicities. Median robust adjusted coefficients of determination (*R*^2^) are indicated by symbols, bars represent 95% confidence intervals (CI). Symbols are referring to Caucasians premenopausal (pre, white circle, *N* = 305), Caucasians post-menopausal (post, black circle, *N* = 352), Arabs (triangle, *N* = 71), Asians (diamond, *N* = 151).

Linear modeling across all three ethnic subgroups revealed that CYP2D6 diplotype showed the highest coefficients of determination for both metabolite endpoints as compared to the five evaluated phenotype classifications inferred from the diplotypes. The explained variability was highest for diplotypes as a predictor of E/DMT with a median *R*^2^ of 68% (premenopausal Caucasians) to 82% (Asians). Likewise, absolute endoxifen concentrations were also best predicted by diplotype, yet to a lesser extent (median *R*^2^: 39–58%; **Figure 2** right and left, respectively). Of the five tested phenotype groupings derived from diplotypes (**Table 1**), TAM4 was superior in its explanatory power for both E (median *R*^2^: 34–52%) and E/DMT (62–65%). Of note, the TAM4 phenotype was adapted by a downgrade of ^∗^10 via introduction of a non-classical *slow* metabolizer phenotype (SM) with ASs halfway between IM and PM (**Table 1**). When compared to TAM2 as the best explanatory phenotype model without modification of ^∗^10 activity, TAM4 was not significantly better in Asians and Middle-Eastern Arabs, however for the prediction of E/DMT, the explanatory *R*^2^ value of TAM4 was slightly superior by 5% in premenopausal (*P* < 0.0001) and post-menopausal (*P* < 0.002) Caucasians. Importantly, the occasionally used codeine specific phenotype classification (Codeine) showed lowest median *R*^2^ of less than 20% for both E and E/DMT in Asians, which significantly differed from TAM2 and TAM4 (*P* < 10^-9^). The two remaining phenotype groupings TAM1 and TAM3 showed intermediary explanatory power for both E and E/DMT, independent of whether ^∗^10 activity was downgraded (TAM3) or not (TAM1).

**FIGURE 3:**
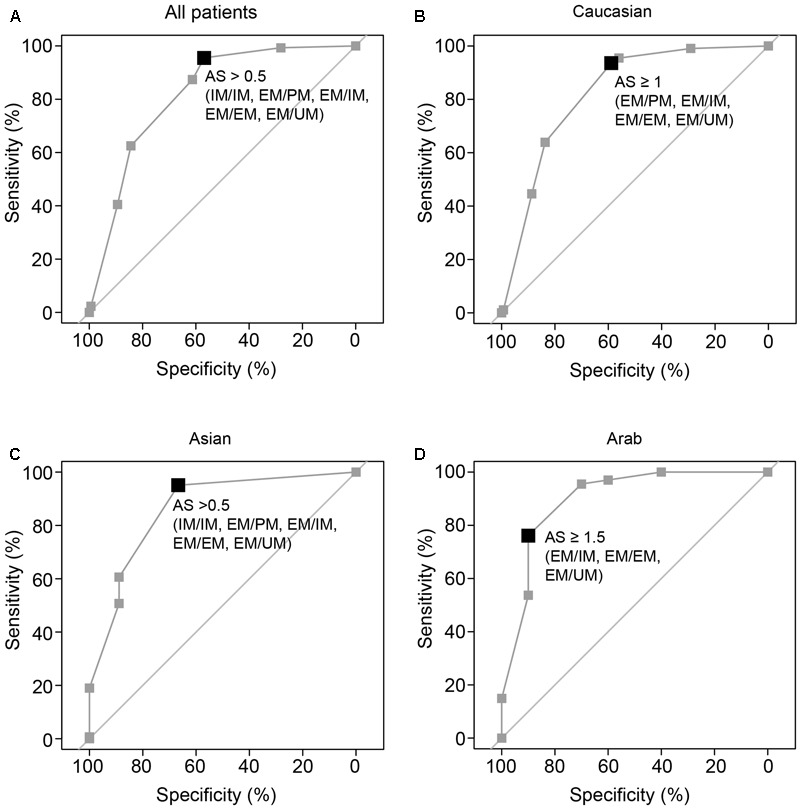
Plot of receiver operating characteristic (ROC) curves for dichotomizing patients into those below and above the clinical threshold endoxifen concentration of 5.9 ng/mL (15.8 nM) based on CYP2D6 diplotype/AS. Bold squares depict the cutoff optimized by Youden index. ROC curves were calculated for all breast cancer patients **(A)** and for ethnic groups in Caucasian **(B)**, Asian **(C)**, and Middle-Eastern Arab **(D)** women. AS definitions were according to Gaedigk et al. (2008), except that EM/PM and IM/IM are distinguished assuming activities of 1 and 0.75, respectively (cf. **Table 2**).

For an evaluation of clinical utility, we applied the single available clinical threshold of 5.9 ng/mL (Madlensky et al., 2011; Saladores et al., 2015) and tested which CYP2D6 diplotype grouping optimally separates patients with higher benefit (above threshold) from those with reduced clinical benefit (below threshold). ROC analyses revealed that overall, patients above the clinical threshold could be largely selected by CYP2D6 diplotypes with AS > 0.5 (IM/IM, EM/PM, EM/IM, EM/EM, EM/UM; median sensitivity 96%; 95% CI: 94–97%; **Table 2** and **Figure 3**). Yet, the specificity across and within ethnicities was moderate (57–90%; **Table 2**) indicating that some patients with endoxifen concentrations lower than 5.9 ng/mL show CYP2D6 AS > 0.5. Approximately 9% of the patients with an AS > 0.5 will not achieve beneficial endoxifen concentrations, a feature which can be mainly attributed to Caucasians (FDR; **Table 2**).

**Table 2:** Classification of CYP2D6 diplotypes predicting patients with plasma endoxifen above the clinical threshold of 5.9 ng/mL.

Cohort	Diplotype (AS)^a^ cutoff	Proportions of patients^f^	Sensitivity % (95% CI)	Specificity % (95% CI)	FDR^g^ % (95% CI)
All	> = IM/IM^b^ (>0.5)	78.5%	96 (94–97)	57 (49–64)	9 (7–10)
Caucasian	> = EM/PM^c^ (≥1)	73.8%	94 (91–96)	59 (51–67)	11 (9–12)
Asian	> = IM/IM^d^ (>0.5)	89.4%	95 (91–99)	67 (33–100)	2 (0–4)
Arabs	> = EM/IM^e^ (≥1.5)	66.2%	76 (66–87)	90 (70–100)	2 (0–6)

*^a^As defined by Gaedigk et al. (2008), except that EM/PM and IM/IM are distinguished assuming a lower activity of the latter. ^b^Includes IM/IM, EM/PM, EM/IM, EM/EM, EM/UM. ^c^Includes EM/PM, EM/IM, EM/EM, EM/UM. ^d^Includes IM/IM, EM/PM, EM/IM, EM/EM, EM/UM. ^e^Includes EM/IM, EM/EM, EM/UM. ^f^Proportion of patients (out of all patients) with AS above cutoff and endoxifen above 5.9 ng/mL. ^g^False discovery rate.*

## Discussion

We re-evaluated a comprehensive data set of CYP2D6 genotypes and TAM metabolite concentrations of breast cancer patients treated with adjuvant TAM to assess the prediction of impaired TAM metabolism by CYP2D6. We applied the power of diplotype-based assignments (Gaedigk et al., 2008) to further refine the discriminatory value of metabolizer phenotype as the most intuitive concept to interpret CYP2D6 polymorphism. To shed light on current controversies on the utility of CYP2D6 for TAM efficacy prediction (Ratain et al., 2013; Brauch and Schwab, 2014; Hertz and Rae, 2016b) standardized genotype–phenotype relationships for the validation of an association between CYP2D6 and impaired TAM metabolism are mandatory.

Currently, the extent to which CYP2D6 determines the up to 20–30 fold (Mürdter et al., 2011) inter-patient variability of plasma endoxifen under standard TAM treatment is poorly characterized. On the assumption that variable TAM metabolism is prognostic for a patient’s response to treatment, it can be argued that drug level monitoring of endoxifen in the first months after treatment start would be straightforward. Yet, since CYP2D6 genotyping requires only a standard molecular biology laboratory and can be used to guide upfront treatment decisions, this approach has been put forward and tested for its predictive value to select patients for TAM dose interventions. Of note, the lack of standardized guidelines to deduce phenotypes from genotype led to the use of a CYP2D6 phenotype classification scheme for impaired Tam metabolism (Fox et al., 2016) that was previously recommended for codeine metabolism (Crews et al., 2014). However, it has become increasingly clear that CYP2D6 variants may exert substrate-dependent effects (Bogni et al., 2005; Gaedigk et al., 2008; Zhou, 2009), and therefore, diplotype specific phenotype data obtained with codeine cannot be extrapolated to other CYP2D6 substrates such as TAM. This functional discrepancy may have important clinical implications as others, based on the inappropriately used codeine scoring concluded that CYP2D6 has no value for the prediction of TAM metabolism (Hertz and Rae, 2016a). Our re-evaluation of existing pharmacogenetic data challenges these findings.

We showed that plasma endoxifen prediction highly depends on the phenotypical grouping of CYP2D6 variant alleles and on the choice of metabolite readout, i.e., absolute metabolite concentrations versus metabolic ratio E/DMT. CYP2D6 diplotypes were superior in predicting endoxifen variability, independent of ethnicity. This was less pronounced when diplotypes were collapsed into fewer class levels (phenotypes), indicating that the effects of functional variants are maximally exploited by a score reflecting the number of null- or reduced-activity haplotypes such as diplotype grouping or AS. Importantly, the codeine-specific phenotype grouping (Crews et al., 2014) poorly predicted CYP2D6-based endoxifen formation. In particular, it was inferior in Asians (median *R*^2^ < 20%) most likely due to a misclassification of abundant IM/IM (^∗^10) diplotypes as EM. From this it follows that CYP2D6 variants act differentially on TAM and codeine substrates, underscoring the need for substrate-specific CYP2D6 genotype–phenotype assessments (Hicks et al., 2014). Within this context, the suggested extra deleterious effect on enzyme function of ^∗^10 compared to other IM alleles (Shen et al., 2007; Hicks et al., 2014) was addressed by downgrading its phenotypic activity and by placing ^∗^10 homozygous patients together with IM/PM diplotypes into a new phenotype category of *slow* metabolizers. A moderate increase of explanatory power (TAM4) by approximately 5% compared to the best explaining phenotype that did not incorporate a ^∗^10 downgrade (TAM2) supports the notion of an increased deleterious effect of ^∗^10 compared to other IM alleles on reduced TAM metabolism. However, given the absence of such an effect in non-Caucasians with a relatively small effect size in Caucasians, the significance of downgrading ^∗^10 for an improved prediction of TAM metabolism must be replicated in larger cohorts and/or meta-analyses.

Our study showed that the metabolite endpoint closely linked to CYP2D6 activity is active metabolite-to-precursor ratio, as more than two thirds of the variability (median *R*^2^: 68–82%) of E/DMT was explained by CYP2D6 diplotype. Notably, a portion of unexplained variability in this study may be related to CYP2D6 alleles that were either not accounted for, e.g., hybrid alleles, or were only partially captured (^∗^2A and other ^∗^2 alleles contributing to EM were genotyped only in Asians), or depend on *cis/trans*-regulatory regions with an influence on CYP2D6 expression that are not yet integrated in biomarker panels. However, the effect of undetected hybrid genes on phenotype is more related to gene duplications and therefore minor (Black et al., 2012), and outliers that strongly differed to expected CYP2D6 function were dispersed over several phenotypic categories (AS ≥ 1) rather than being limited to EM. Therefore, it is plausible to postulate the existence of additional genetic loci, that, similar to a previously described enhancer (Wang et al., 2013, 2015) influence CYP2D6 expression in a modest number of patients, a hypothesis which needs to be further investigated. Moreover, plasma endoxifen variability was predictable to a lesser extent (39–58%) compared to the highly CYP2D6-dependent E/DMT. Therefore, factors other than CYP2D6 genetics may account for the unexplained portion of endoxifen variability such as non-compliance, CYP2D6 inhibitor use, environmental factors, and other cytochrome P450 isoenzymes including CYP3A phenotype (Teft et al., 2013; ter Heine et al., 2014). Although our genetic model did not incorporate CYP3A4 activity predicted by the ^∗^22 variant (Wang et al., 2011; Teft et al., 2013; Antunes et al., 2015), fluctuations in plasma levels of endoxifen precursors (4OH-TAM, desmethyl-TAM) were accounted for by adjusting for CYP2C9 and CYP3A5 variants, while strong CYP2D6 inhibitor use was low to absent in the majority of patients. Thus, our E/DMT-based translations of diplotypes into metabolizer phenotypes TAM4 and TAM2 with or without downgrading of ^∗^10 activity, respectively, capture most of the variability attributable to CYP2D6 and are superior to previous CYP2D6 metabolizer assignments such as TAM1 (Irvin et al., 2011; Hertz et al., 2015) and the codeine score (Fox et al., 2016).

Although our study does not provide direct data for clinical outcome prediction, an endoxifen threshold concentration of 5.9 ng/mL useful to predict breast cancer recurrence risk during TAM therapy (Madlensky et al., 2011) is predictable by CYP2D6. Overall, a genotyping test discriminating patients based on an AS cutoff of 0.5 revealed a greater than 95% sensitivity to predict whether patients will have beneficial endoxifen concentrations. This simplified test interpretation may help clinicians to reassure their upfront treatment decisions, i.e., standard TAM above threshold, versus TAM dose adjustment or AI choice around and below threshold. Given our observation of a moderate specificity, e.g., in Caucasians, an approach of maximizing specificity at the cost of decreased sensitivity to avoid the risk of false-positive CYP2D6 testing could be a strategy which needs further investigation based on independent cohorts. Moreover, the increased FDR in Caucasians (11%) compared to Asians and Middle-Eastern Arabs (2%; **Table 2**) point to a modest proportion of TAM treated patients tested positive but having sub-therapeutic endoxifen concentrations, which would bear a risk of undertreatment. Together with the fact that genotyping does not provide information on treatment adherence, a combination of upfront genotype-informed treatment allocation followed by therapeutic blood monitoring for active metabolite concentrations therefore appears promising to personalize TAM treatment use.

In summary, we provided an improved algorithm to predict CYP2D6-dependent impaired TAM metabolism from genotype underscoring its essential role in drug bioactivation toward endoxifen independent of age and ethnicity. Clinical evaluation of standardized TAM-specific CYP2D6 activity assignments may shed new light on linking impaired endoxifen formation with TAM outcome prediction aiding the selection of patients for TAM dose increase or AI treatment.

## Author Contributions

WS, SW, TM, MS, and HB designed and performed the study. DE, BE, BC, CK, AT, and NZ recruited study participants and provided specimens for analyses. WS, SW, TM, ES, ME, MS, and HB are responsible for data interpretation, critical review, and manuscript preparation. All authors approved the manuscript.

## Conflict of Interest Statement

The authors declare that the research was conducted in the absence of any commercial or financial relationships that could be construed as a potential conflict of interest.

## Footnotes

1 www.r-project.org
